# Investigating PLGA microparticle swelling behavior reveals an interplay of expansive intermolecular forces

**DOI:** 10.1038/s41598-021-93785-6

**Published:** 2021-07-15

**Authors:** Crystal E. Rapier, Kenneth J. Shea, Abraham P. Lee

**Affiliations:** 1 Department of Biomedical Engineering, University of California-Irvine, Irvine, CA USA; 2 Department of Chemistry, University of California-Irvine, Irvine, CA USA

**Keywords:** Materials science, Drug delivery, Biomedical engineering, Chemistry, Polymer characterization

## Abstract

This study analyzes the swelling behavior of native, unmodified, spherically uniform, monodisperse poly(lactic-co-glycolic acid) (PLGA) microparticles in a robust high-throughput manner. This work contributes to the complex narrative of PLGA microparticle behavior and release mechanisms by complementing and extending previously reported studies on intraparticle microenvironment, degradation, and drug release. Microfluidically produced microparticles are incubated under physiological conditions and observed for 50 days to generate a profile of swelling behavior. Microparticles substantially increase in size after 15 days, continue increasing for 30 days achieving size dependent swelling indices between 49 and 83%. Swelling capacity is found to correlate with pH. Our study addresses questions such as onset, duration, swelling index, size dependency, reproducibility, and causal mechanistic forces surrounding swelling. Importantly, this study can serve as the basis for predictive modeling of microparticle behavior and swelling capacity, in addition to providing clues as to the microenvironmental conditions that encapsulated material may experience.

## Introduction

The copolymer poly(lactic-co-glycolic acid) (PLGA) is an FDA approved biocompatible material that is used for a variety of clinical and therapeutic applications ranging from intravenous and pulmonary drug delivery vehicles^1–4^, anti-inflammatory implantable devices^5,6^, to suture, scaffold, and graft materials^7–9^. PLGA is degradable and bioabsorbable, making it highly desirable as a drug delivery device specifically in the form of micro and nanoparticles. In order for PLGA microparticles to be efficient drug delivery devices, it is critical to understand the physiochemical properties of PLGA as they help in the prediction and modification of polymer performance as it relates to drug stability and subsequent release.

**Figure 1:**
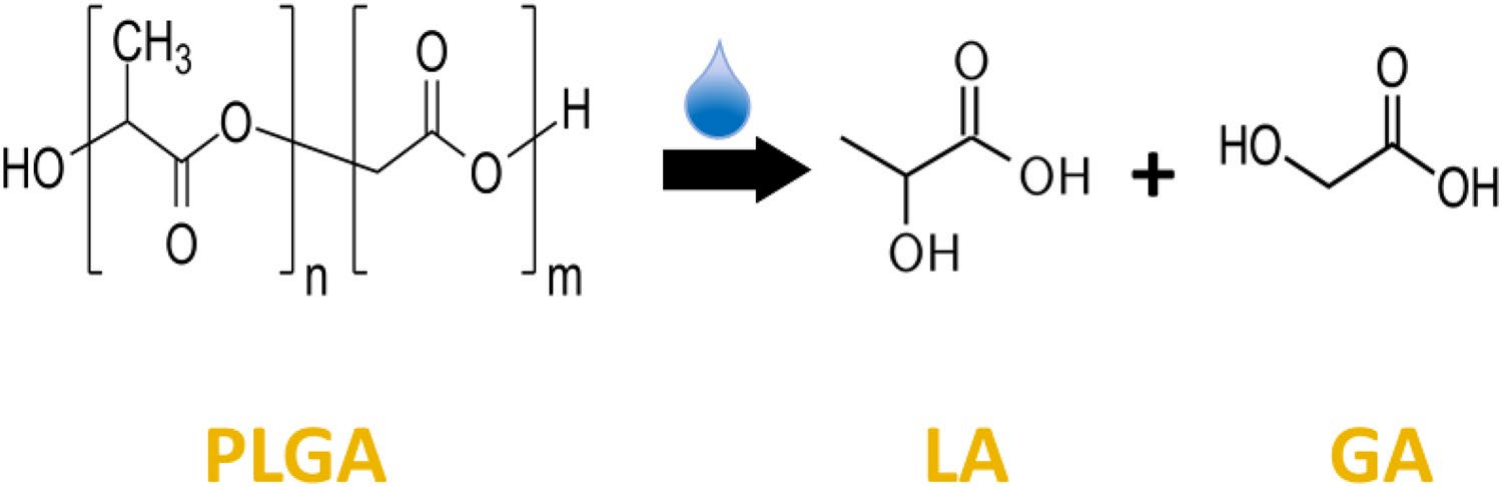
Chemical structure of PLGA polymer. PLGA is hydrolytically cleaved into lactic acid (LA) and glycolic acid (GA) monomers.

PLGA degrades mainly via hydrolytic scission of the ester linkages between its lactic acid (LA) and glycolic acid (GA) moieties (Fig. 1). The longevity of PLGA can be altered by changing the polymer length (molecular weight) and its end-capping—where another end group is substituted for the native carboxyl terminal groups. PLGA is a bulk (heterogeneous) eroding and thus not a surface (homogeneous) eroding polymer^10,11^. Overall, polymer degradation and erosion are controlled by polymer crystallinity, lactic to glycolic acid molar ratio, molecular weight (mw), polymer end-capping, water absorption, hydrolysis, pH, and glass transition temperature (T_g_). Degradation is marked by loss in mw followed by erosion which is marked by loss in overall weight of the polymer. Loss in mw is also accompanied by a reduction in the T_g_—the temperature at which the polymer transitions from a rigid glassy state to a more relaxed and flexible rubbery state^11,12^.

Whether in slab, disk, or microparticle form, PLGA is said to have size dependent autocatalytic capabilities in which the center of a PLGA matrix degrades faster than the rest of the matrix at or toward its surface due to an accumulation of trapped acidic oligomers^13–16^. This accumulation of oligomers results in the development of a highly acidic core at the center of the microparticle matrix. The acidic core microenvironment of PLGA microparticles has been evaluated^17–19^; however, the first study to visualize, measure, and monitor the change in internal pH of individual drug-free microparticles over a period of time was done by the Langer group^14^. They used a direct approach to quantitatively evaluate the spatial distribution of the intraparticle acidic environment under physiological conditions (37 °C, phosphate buffered saline (PBS) solution). They visually observed the development of a highly acidic internal core within PLGA microparticles as low as pH 1.5 in some cases. They reported that after a period of 15 days, the acidic core dissipated. We observed microparticles beginning to expand in size after 15 days of incubation under the same conditions and continue to substantially expand thereafter.

Although not discussed at length, PLGA matrices, such as slabs and disks have been shown to absorb water (swell) and subsequently increase in size (expand) at or above physiological temperature conditions^10,13,20^. While swelling does occur, it is largely ignored in drug delivery system publications and mathematical modeling^16^. A typical drug release study involves removing and measuring the supernatant of a bulk-made heterogeneous polydisperse microparticle sample^21–26^. Despite the occasional Scanning electron microscope (SEM) image, particles are not viewed throughout the study for in-depth analysis of particle behavior, leaving particle swelling to be unnoted. In recent years, few studies have investigated the swelling of individual PLGA microparticles^27,28^. However, these studies evaluated swelling using inherently unreproducible polydisperse microparticle samples. The particles encapsulated acidic or basic bioactive compounds which alters particle behavior; and, the studies do not include a prolonged evaluation of swelling behavior.

Our study complements and extends the work of the Langer group and others by analyzing microparticle swelling behavior in a robust, high-throughput manner and by discussing the physiochemical changes contributing to the phenomenon of PLGA microparticle water uptake and substantial expansion. Using the same microparticle sizes examined by the Langer group (~ 14–40 µm), the authors of this article use a prolonged 50-day study of 15 monodisperse native microparticle samples without the addition of drugs, proteins, or excipients into the polymer matrix to answer questions surrounding swelling and expansion such as: onset, swelling index, size dependency, and reproducibility. The use of native unmodified microparticles can help elucidate release mechanisms of the natural polymer. In the end, our study will provide swelling behavior profiles, a timeline, and an explanation as to the probable mechanism behind the profile trends. This study can also serve as a canonical foundation for predictive modeling of microparticle swelling capacity and volumetric change. Predicting the final volumetric change has several advantages, e.g., knowing the maximum size that swollen microparticles can achieve would help predict what blood vessels the particle can safely travel and ultimately anticipating what tissues the particle can target and traverse for drug delivery. Size is also critical when dealing with cells such as macrophage that encounter drug delivery microparticles along common entry routes such as the lungs and the blood. The phagocytic, proinflammatory, and foreign body response of macrophage is largely dependent on particle size^29–32^. In addition, water uptake is an important physiochemical process that ultimately influences PLGA microparticle drug release kinetics^33–35^; and, therefore must be understood in order to develop a truly “controlled” drug delivery system.

## Results and discussion

### Fabrication of PLGA microparticles

**Figure 2:**
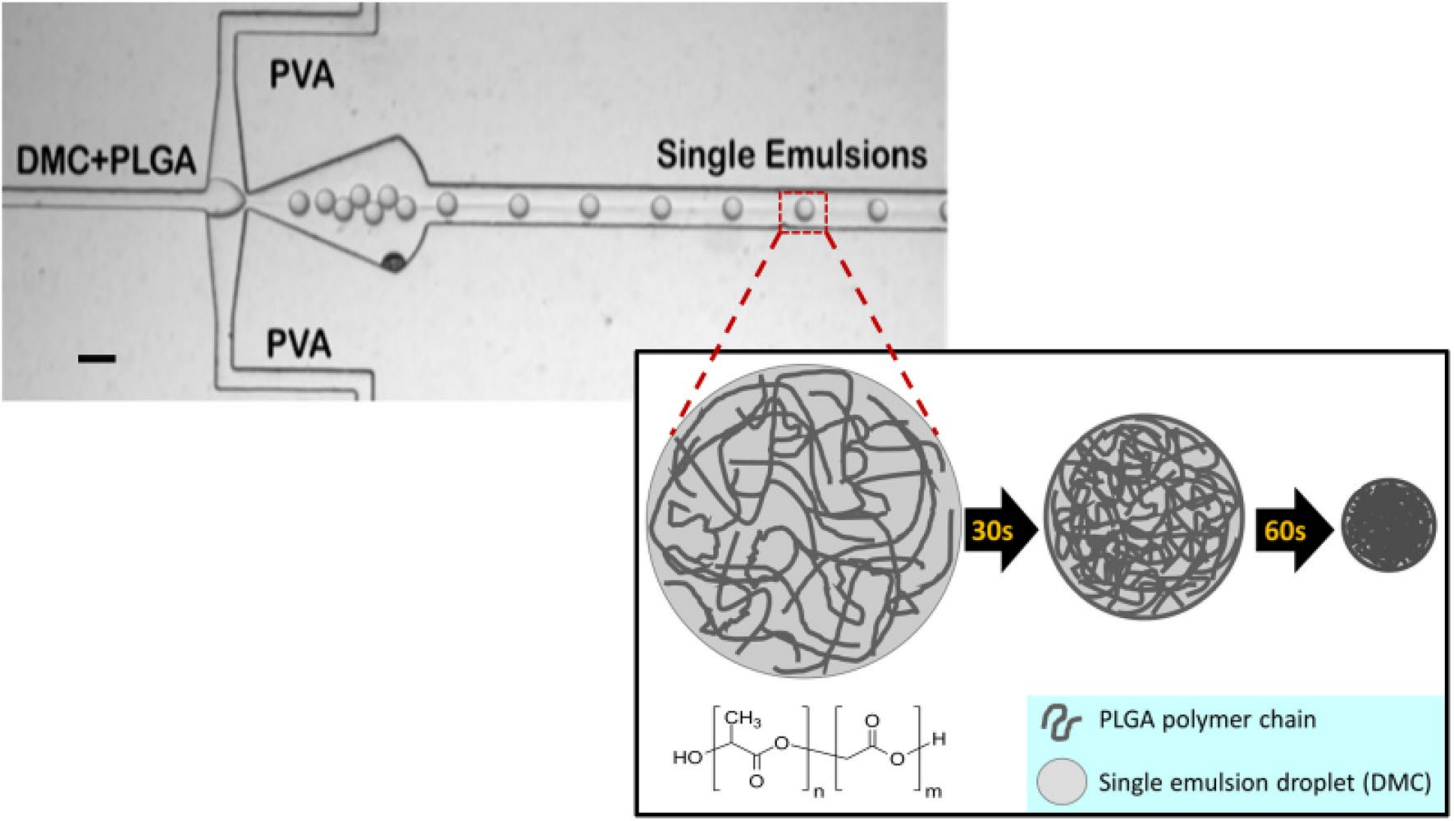
Image of the microfluidic device generating single emulsions (embryonic microparticles). Device design includes one flow focusing junction which creates oil in water (O/W) emulsions by dispersing a PLGA-DMC solution into a 1 wt% polyvinyl alcohol (PVA) continuous phase solution. Direction of flow is from the left to the right. Scale bar is 40 µm. Inset is a schematic of DMC solvent extraction from a single emulsion droplet, yielding one PLGA microparticle. Embryonic microparticles consist of organic solvent, DMC, and PLGA. Complete droplet transformation from an embryonic state into a microparticle occurs after 60 s.

Monodisperse PLGA microparticles were successfully created using microfluidics. Microparticles were prepared using a standard oil-in-water (O/W) single emulsion solvent extraction process. Our device design for single emulsion generation includes a single flow-focusing junction where two channels containing a dispersed and continuous fluid phase intersect at a pressure reservoir^36^. The dispersed phase consisted of PLGA dissolved in the non-toxic organic solvent, dimethyl carbonate (DMC). Whereas 1 weight percent (wt%) polyvinyl alcohol (PVA) solution served as the continuous phase solution (Fig. 2). The intersection of the two fluid phases produces discrete droplets (single emulsions) from the perturbation of the discrete phase fluid thread by the continuous phase. The solvent exchange (diffusion) and subsequent extraction of DMC from a single emulsion droplet leaves PLGA polymer chains behind to condense into a single microparticle (Fig. 2). Microparticle formation from emulsion droplets (embryonic microparticles) occurred within 1–2 min.

It is important to selectively treat channels to increase hydrophilicity and subsequent flow shearing efficiency during droplet production^37^. Briefly, the continuous phase channel was selectively treated with PVA to render it hydrophilic, while the dispersed phase remained untreated and hydrophobic. This strategy allows the dispersed phase to be sheared into discrete droplets at the flow-focus region. The selective treatment of the continuous phase channel also helps to prevent wetting of the DMC + PLGA solution to the channel walls. Wetting of this solvent is irreversible and results in the inability to form droplets. Microparticles were collected using a pipette or by connecting Teflon tubing to the outlet of the microfluidic device. Microparticles were deposited into a microcentrifuge tube containing PBS for testing.

### Characterization of PLGA microparticles

**Figure 3:**
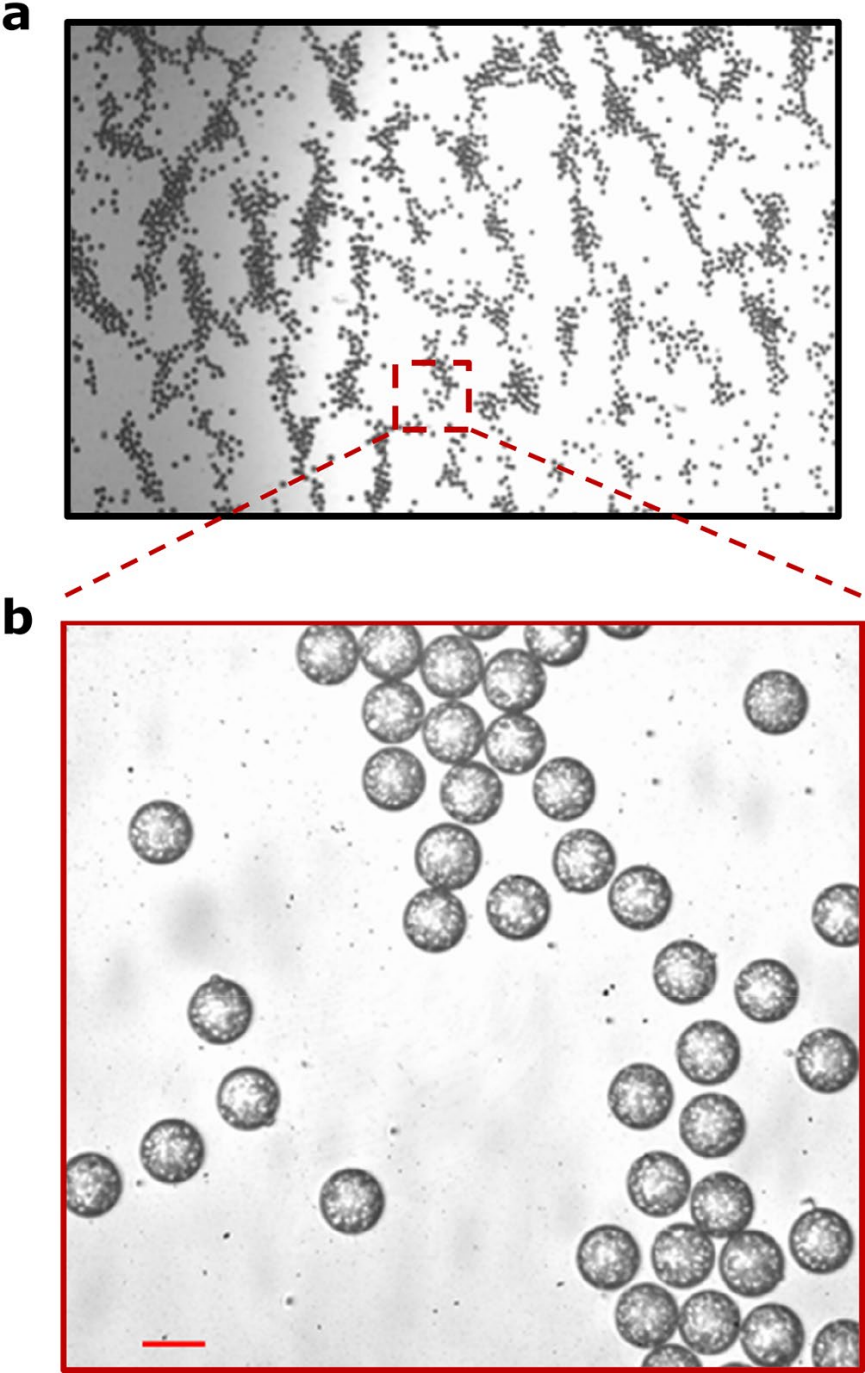
Magnification of 29 µm microparticles demonstrating the monodispersity (PDI = 1.6%) and morphology. ×4 magnification (**a**) and ×40 magnification (**b**) on an inverted microscope. Scale bar is 29 µm.

**Figure 4:**
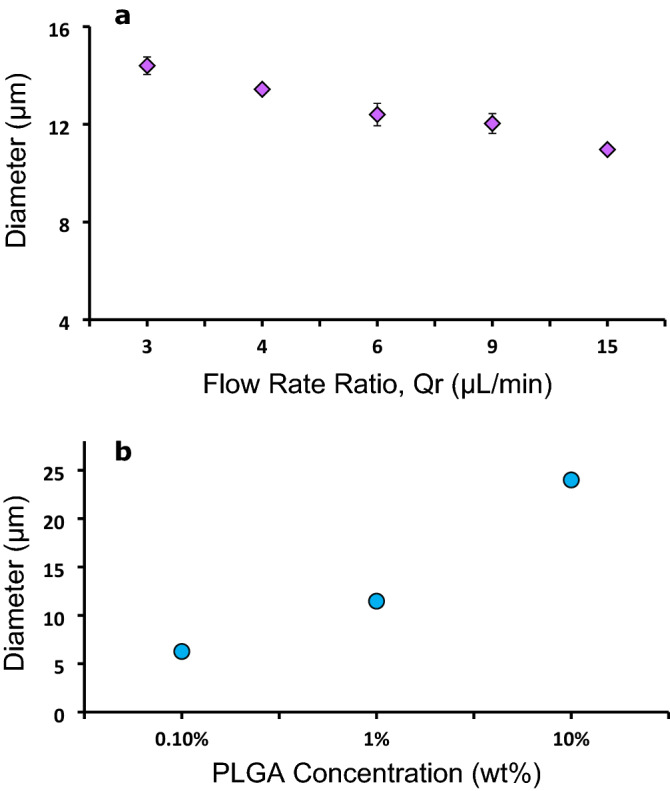
Microfluidic modulation of particle size. (**a**) Microparticle size range generated using 1 wt% PLGA at different flow rates. Q_r_ is the flow rate ratio of continuous to dispersed phase. (**b**) Modulation of particle diameter using various concentrations of PLGA at a Q_r_ of 9. n ≥ 3 experiments for (**a**) and (**b**).

For swelling study purposes, microparticles ranging in size from 11 to 44 µm were produced using microfluidic techniques. Figure 3 demonstrates the morphology and monodispersity of generated particles. Polydispersity index (PDI) was used to measure the consistency of microparticle size within each sample population. The PDI (σ) was calculated by Eq. (1):

1 $$\sigma = \frac{\delta }{{D_{A} }} \times 100\% ,$$

where δ is the standard deviation and D_A_ is the average particle diameter. Results in Table 1 demonstrate that the microparticles generated were nearly monodisperse with polydispersity indices between 0.7 and 2.7 percent. Figure 4 shows the sizes of particles that can be generated through different flow rates of the continuous and dispersed phases along with different percent weight concentrations of PLGA. Flow rates of 3, 4, 6, 9, and 15:1 continuous (Q_c_) to dispersed phe (Q_d_) generate 1 wt% PLGA microparticles with an average diameter of 14–11 μm respectively. These flow rate ratios (Q_r_) resulted in highly reproducible particle sizes with a standard deviation less than 0.5 with n ≥ 3. Continuous phase flow rates below 3 μL/min for this flow focusing design did not generate consistently sized droplets and subsequent particles due to the PLGA + DMC dispersed phase wetting the PDMS. Flow rates higher than 15:1 μL/min did not create any sizable change in droplet or subsequent particle diameter. The flow rate range (threshold) for the microfluidic device depicted in Fig. 4a was 3:1–15:1 µL/min. We used various device designs with different thresholds to generate microparticles. Flow rates for particle batch production are included in Table 1.

**Table 1:** Characteristics and production conditions of tested microparticles.

Flow rate (µL/min)	D_o_ (µm)^a^	σ (%)	D_t_ (µm)	SI (%)
25:1	11 ± 0.2	2.1	17 ± 0.2	49
4:1	14 ± 0.4	2.7	21 ± 0.1	51
9:1	23 ± 0.6	2.7	43 ± 0.3	83
7:0.5	30 ± 0.7	2.3	56 ± 0.5	83
4:1	44 ± 0.3	0.7	79 ± 1.4	82

^a^Mean size of the investigated microparticles ± standard deviation (n = 30).

#### Effect of PLGA concentration on particle size

In addition to adjusting flowrates and using various device designs, microparticle diameters can be controlled by utilizing various percent weight concentrations of PLGA. There is a threshold or a set range of microparticle sizes that can be generated when relying on the flow rates alone. Therefore, we adjusted the PLGA weight percent concentration within a flow rate threshold to produce a wider range of particle sizes. For example, a flow rate ratio of 4:1 µL/min continuous to dispersed phase and 10 wt% PLGA produces 29 µm particles. Whereas, using the same flow rate of 4:1 µL/min and a 1 wt% PLGA concentration produces 13 µm particles. Figure 4b simply illustrates this using a single flow rate of 9:1 µL/min and PLGA concentrations of 0.10–10 wt% to produce particles with average diameters of 6–24 µm respectively. In addition, altering device design dimensions along with PLGA concentration affect the size range of microparticles.

### In vitro swelling study

Microfluidically generated microparticles were studied to observe the onset of swelling, its duration, and behavior. For the swelling study, five different sizes of microparticle samples (sample populations) with initial diameters of 11–44 µm were produced. Triplicates were made of each size, totaling 15 samples. Microparticles were suspended in PBS solution, pH 7.4, and incubated continuously for 50 days under physiological conditions (37 °C). Aliquots of each triplicate sample were removed for microparticle measurement on days 0, 5, 10, 15, 30, 40, and 50. On average, 25 microparticles were measured per aliquot.

**Figure 5:**
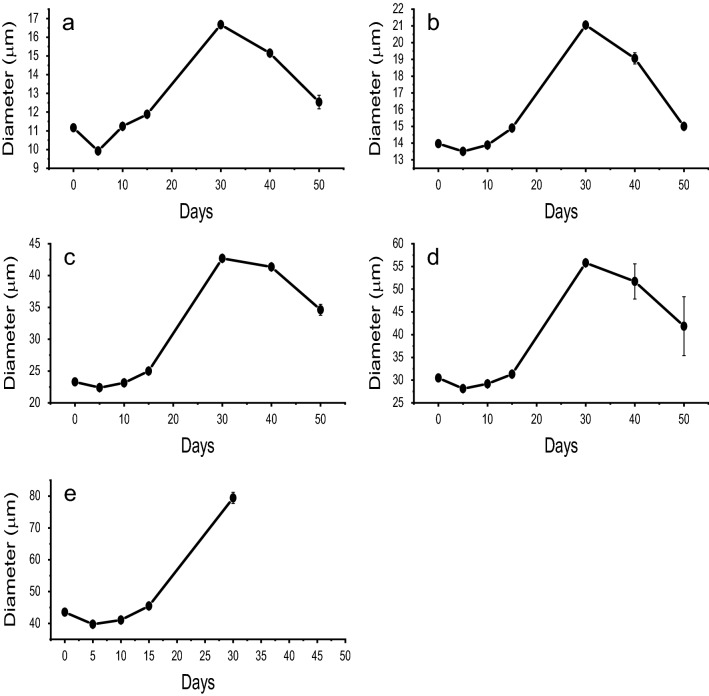
Swelling profiles of various microparticle population sizes: 11 µm (**a**), 14 µm (**b**), 23 µm (**c**), 30 µm (**d**), and 44 µm (**e**).

**Figure 6:**
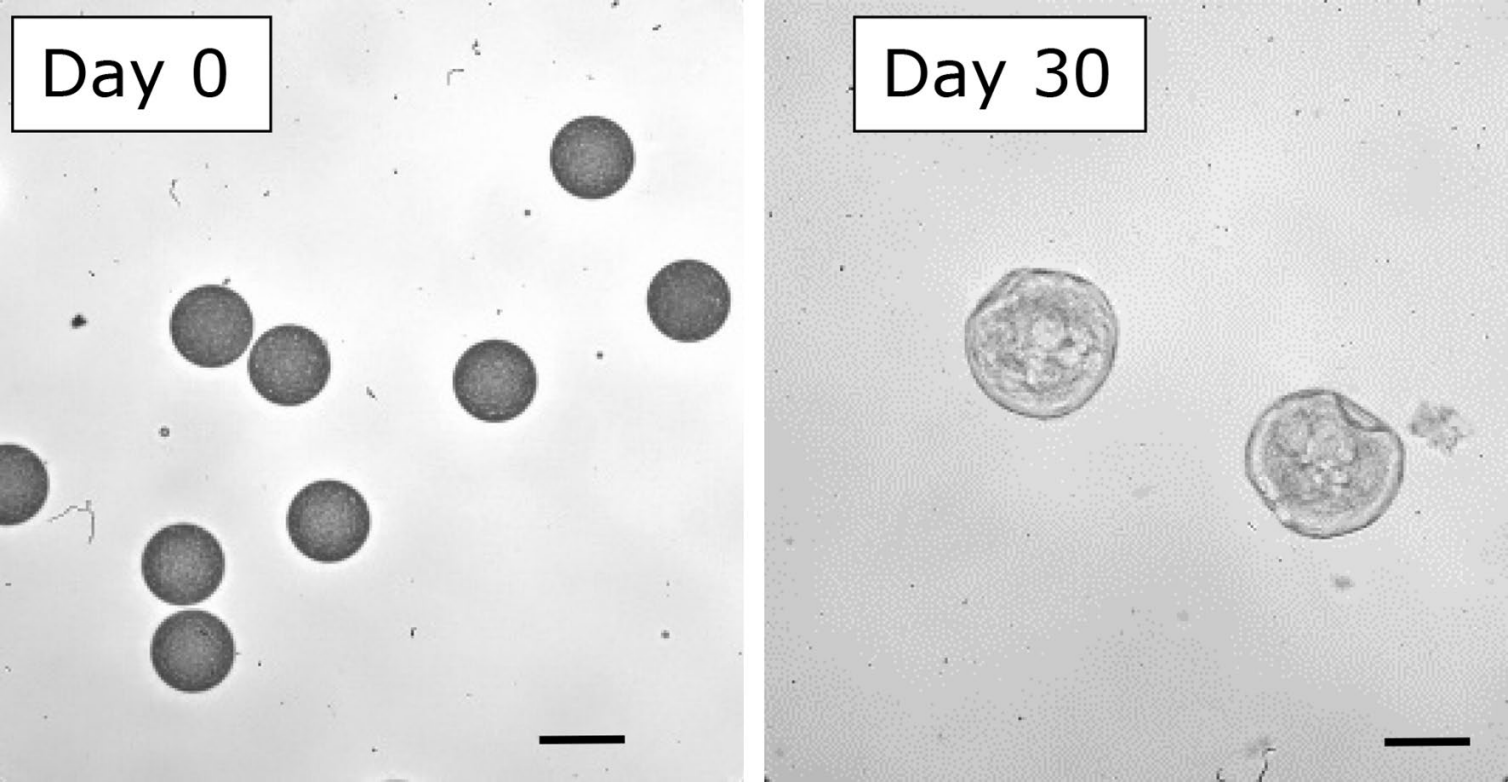
Microparticle morphological changes over time. Images taken at ×40 magnification comparing size and morphology of original 44 µm microparticles on day 0 and swollen microparticles (79 µm) on day 30. Scale bar is 44 µm.

**Figure 7:**
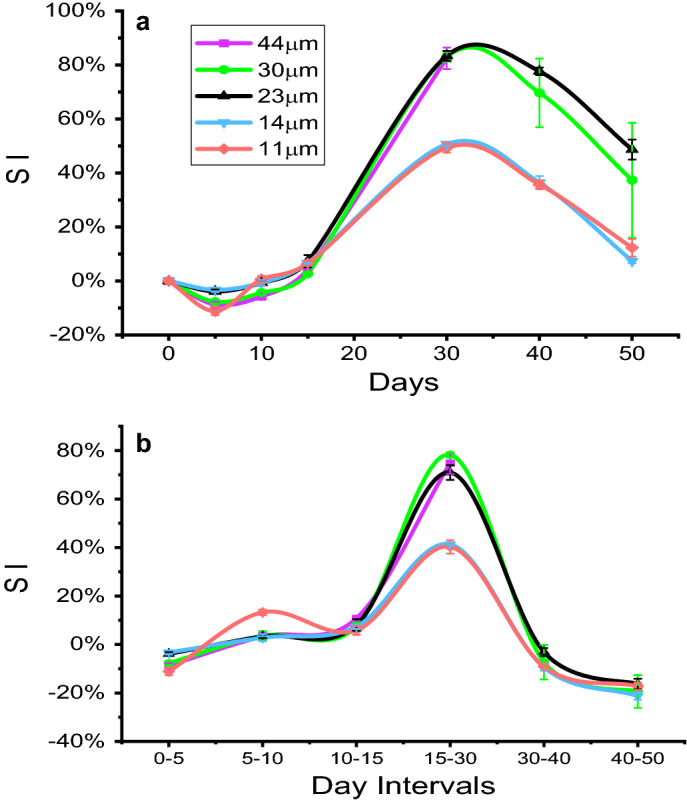
Swelling kinetics. (**a**) Complete swelling index (SI) changes from day 0. (**b**) Swelling changes between each incubation time interval.

Figure 5 is a graph of microparticle swelling behavior over time (swelling profile). Microparticles appear to decrease in size (shrink) after 5 days of incubation. After 10 days of incubation microparticles return near their original size and between 15 and 30 days began to substantially increase in diameter (swell). All microparticle sample populations reached a maximum diameter by day 30, declining in size thereafter. The 44 µm sample populations were too irregular due to degradation after more than 30 days and were not included for days 40–50 in the graphs. Table 1 shows the swelling index (SI) of the various sizes of microparticle populations. SI provides an indication of microparticle capacity to absorb water. SI was calculated according to Eq. (2):

2 $$Swelling~\,Index = \frac{{D_{t} - D_{0} }}{{D_{0} }} \times 100\% ,$$

where D_t_ represents the maximum diameter achieved during the 50-day incubation period and D_o_ is the initial diameter of microparticles. Swelling indices of up to 83% were achieved by the microparticles. Results show that swelling correlates with size. Microparticles < 15 µm diameter had a SI of 49–51%, whereas microparticles > 15 µm diameter achieved a SI of 82–83%. Images comparing the diameters of microparticles on day 0 verses day 30 are shown in Fig. 6. These microparticles were initially 44 µm on day 0 and increased to a diameter of 79 µm by day 30. A detailed analysis of swelling kinetics is presented in Fig. 7. Figure 7a is the complete SI produced over the 50-day course of the study. By day 5, a division of the microparticles into a group composed of 11, 14, and 23 µm populations and a second group composed of 30 and 44 µm populations emerged. Interestingly, the 11 µm microparticle populations showed the greatest fluctuation between day 0 and day 10, after which the microparticles join and follow the 14 µm microparticle population trend. After 15 days—a juncture where all the microparticles had similar swelling indices—the 23 µm microparticles followed the trends of the larger 30 and 44 µm particle populations. It is possible that the microparticles are grouped according to relative sizes with particles smaller than 23 µm following the lower trend. Additional studies with a broad range of microparticle sizes would help clarify this assertion. Figure 7b shows the rate of swelling between each incubation time interval. The 11 µm microparticles had the fastest rate of change between each time interval, joining the other particle profiles by day 15. Again, two distinct groups emerge after 15 days with 11 and 14 µm populations following the same swelling rate trend and the 23, 30 and 44 µm populations following another. The larger microparticles shrank drastically from their peak diameter, joining the smaller microparticles in terms of shrinkage rates after 30 days. Taken together, Fig. 7a,b suggest a distinction between the swelling trends of large (> 15 µm) and small (< 15 µm) microparticles.

### Microparticle behavior as it relates to the swelling profile and other intraparticle studies

To offer an explanation as to the mechanistic physiochemical changes that occur to cause microparticle swelling, we compared our data to the Langer group’s visual intraparticle study data of ~ 14–40 µm particles. The following selected results were reported by their observation of the internal environment of PLGA microparticles suspended in unchanged PBS buffer at 37 °C^14^: (1) A concentration gradient develops with pH values decreasing toward the central core region of the microparticles after a short incubation period; (2) The size and pH of the acidic core region depends on the diameter of the microparticle, i.e., large microparticles developed a more acidic core verses smaller ones; (3) The polymer weight-average molecular weight dropped precipitously; whereas, the dry weight of the microparticles remained the same during 15 days of incubation; and (4) The acidic core was reported to eventually dissipate after 15 days—suggesting acidic degradation products slowly escaped out of the particles through a diffusion-controlled mechanism. Taken together, our data, and other reports present the following narrative about the mechanisms behind the delayed substantial swelling of PLGA microparticles.

The extraction/diffusion of DMC from single emulsion droplets leaves PLGA polymer chains behind to condense into a microparticle (Fig. 2). We observed further shrinkage of all microparticles on day 5 of incubation. This could be the result of high polymer chain entanglement reinforced by intermolecular bonds between the chains. Among such molecular forces would be the energy favorable rearrangement and aggregation of polar and nonpolar regions within the thermally relaxed polymer. Namely, the lactiderich regions being more hydrophobic than the glycolide regions would tend to self-associate. Furthermore, shrinkage could occur from energy-favorable dimers forming between polymer chain carboxyl terminal groups. These dimers are stabilized with increasing polymer chain length and hydrophobic bonds between hydrophobic regions in aqueous solutions^38,39^. The complete diffusion of DMC out of the newly formed microparticle produces an osmotic pump. Water or PBS buffer will slowly infiltrate toward the center of the particle to compensate for the osmotic pressure difference. PLGA absorbs a penetrant such as water according to the “case-II diffusion model”^20,40^. The case-II diffusion model describes the penetration of water (swelling) as being driven by osmotic pressure which relaxes to zero once the penetrant surface volume fraction reaches equilibrium. The model states that the rate-controlling factor is the viscosity of the polymer^40^.

**Figure 8:**
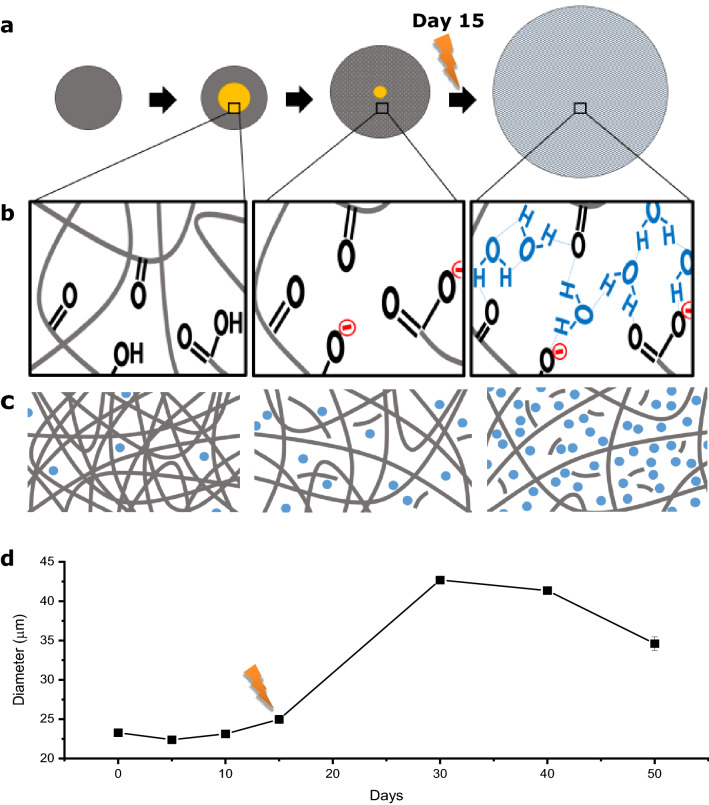
Interplay of expansive intermolecular forces. (**a**) Schematic of microparticle swelling over time. Grey circles represent microparticles. Yellow circles represent the development of an acidic core region and the lightning bolt signifying point of substantial swelling. (**b**) Insets represent the hydrolysis, degradation, hydration shell and swelling that occurs over time in terms of active chemistry. (**c**) The corresponding general view of water ingress over time, subsequent hydrolysis of polymer, and ultimate expansion of relaxed, ionized polymer chains (grey lines represent PLGA polymer chains and blue dots represent water molecules). (**d**) Swelling profile timeline as it correlates with the schematic (taken from 23 µm population data).

Hydrolysis of PLGA polymer chains occurs as a result of water infiltration, marked by a decrease in Tg—a measure of chain mobility/flexibility^19,41,42^; mw—a measure of chain length^11,43^; and ultimately a loss in polymer mass^10^. Through random and end chain scission, hydrolysis generates protons by exposing more acidic carboxyl and hydroxyl groups at the ends of newly formed PLGA oligomers and monomers (Fig. 1). The Langer group reports this degradation occurs over the 15-day incubation period as seen by the precipitous drop in polymer weight-average mw. Erosion is evidenced by a change in the dry weight of the microparticle which reportedly remained constant for 15 days. The short chain acids become trapped in the polymer matrix decreasing the internal pH of the microparticle. This process starts after a couple of days of incubation and continues up to 15 days, as seen in various visualization data^14,19,44^. During this time pH, mw, Tg, and particle mass continue to drop due in part to the microparticle’s autocatalytic cyclic activity whereby hydrolysis creates more acids which in turn act as additional catalysts for hydrolysis, accelerating polymer degradation. Autocatalysis is concentrated in the center or core of the PLGA microparticle matrix due to an accumulation of trapped acidic oligomers^13–16^ and its activity increases with increasing particle diameter^12,14,18,19^. Furthermore, the volume of the autocatalytic acidic core depends on the size of the microparticle^14^. The increasing number of oligomers (acidic degradation products) formed within the center of the microparticle are sequestered until a time when the microparticle has eroded enough to generate pores for escape. Langer group reported that the highly acidic core region dissipated after 15 days showing evidence that acidic degradation products escape via a diffusion-controlled mechanism. With increased pore formation, water fills in the void spaces left behind by the acidic oligomers to satisfy diffusive forces of the microparticle’s induced osmotic pump—helping to neutralize the core region. Figure 8a schematically shows microparticle development of an acidic core and its dissipation as a microparticle expands. Figure 8b,c depict the chemistry and generalized view behind the progression in Fig. 8a. Figure 8d is a profile of the 23 µm microparticle population temporal trend as it correlates with the physical transformation and chemical change schematics of Fig. 8a–c. The 23 µm population profile in Fig. 8d is used as a general swelling profile for all microparticles as the overall trends are similar between all microparticle populations tested.

Not only does water ingress fill in the spaces left by the escaping oligomers and help neutralize the core pH, they also cause thermal and mechanical changes to the microparticles due to a process called plasticization (Fig. 8b). Water is an excellent plasticizer and solvent of PLGA. As a plasticizer, water favorably interacts with and increases polymer chain mobility by stretching them out^41^. The marked decrease in microparticle Tg is a result of more mobile water-polymer bonds replacing less mobile polymeric intermolecular H-bonds^42^, relaxing PLGA from a glassy to a rubbery state. Rates of diffusion and relaxation increase with a rubbery state above Tg^19,40,45^. In addition, heat increases polymer degradation^46^ and water ingress—as water molecules within the buffer have more kinetic energy^20,47^. With the core region increasing its pH to a more neutral state, the carboxyl terminal groups in the polymer will become deprotonated and subsequently ionically charged forming carboxylate ions when pH ≫ pKa. According to the Henderson–Hasselbalch equation (Eq. (3)), carboxyl groups with a pKa of ~ 4 will be 99% in ionized form at a pH of 7.

3 $${\text{pH}} = {\text{pK}}_{{\text{a}}} + {\text{log}}\left( {\frac{{\left[ {conjugate~\,base} \right]}}{{\left[ {weak~\,acid} \right]}}} \right).$$

Based on our findings, we hypothesize that the accumulation of carboxylate ions leads to the electrolytic repulsive forces that cause the relaxed polymer to physically expand, creating more space in the polymer matrix, i.e., a volumetric change (Fig. 8b). This physical change influences water uptake. Water ingress is further increased by its attraction to the newly formed carboxylate ions through ion–dipole hydrogen bonds. Hydrogen bonding can create a hydration shell around the carboxylate ions effectively increasing and stabilizing the spaces created by the electrolytic repulsion^48,49^. We found that particles substantially increase in size after 15 days of incubation coinciding with core neutralization data^14^. Therefore, we conclude that it takes approximately 15 days for the particle to degrade enough to create clear paths of diffusion for the small oligomers and acidic monomers to escape the core and for the water/buffer to fully diffuse in to neutralize the core region. Thus, changing the carboxyl groups to carboxylate groups, ultimately leading to electrolytic repulsion and microparticle volumetric change.

**Figure 9:**
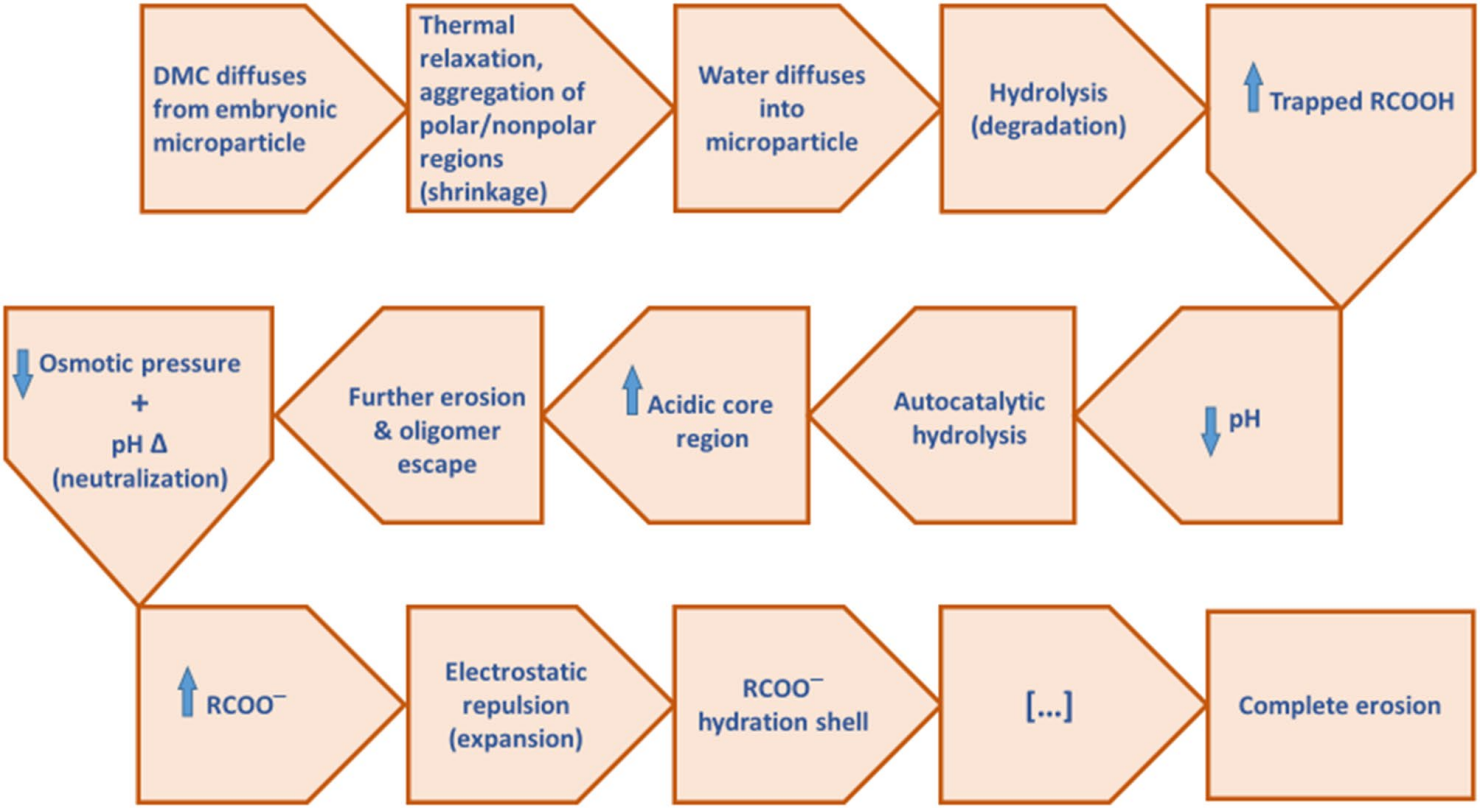
Proposed general course of intermolecular forces behind PLGA microparticle swelling behavior. Arrows represent an increase or decrease of corresponding element and Δ represents change.

Our findings demonstrate swelling continues for another 15 days or so, peaking at 30 days for all microparticle sizes tested. Following Table 1, it appears that the swelling capacity of microparticles correlates with their original size and likely the size of the acidic inner core region, which is also corroborated by size-dependent autocatalysis theories and Langer group data depicting the size and pH of the acidic core region depended on the diameter of the microparticle. This suggests that volumetric change/swelling is linked to changes in pH. The flowchart in Fig. 9 describes the proposed general course of molecular forces behind PLGA microparticle swelling behavior. This does not imply that the series of events leading to microparticle expansion is completely linear or absolute. This course of events is merely a suggested narrative based on results herein and other comparable PLGA studies.

## Conclusion

In this study, monodisperse microparticles with sizes ranging from 11 to 44 µm were prepared using a standard O/W single emulsion solvent extraction process. Microparticles were incubated at 37 °C and observed for up to 50 days. The onset of swelling, determined by an increase in diameter, occurred between 5 and 10 days after incubation with a substantial change after 15 days. Swelling peaked at 30 days for all microparticle sizes tested, after which the microparticles decreased in size. Microparticles < 15 µm diameter had a SI of 49–51%, whereas microparticles > 15 µm diameter achieved a SI of 82–83%. Results demonstrate Sl correlates with the original size of microparticles. Swelling indices between each incubation interval shows somewhat consistent swelling behavior between the microparticles suggesting a distinction between large and small microparticles. The smallest microparticles tested (11 µm) showed the fastest rates of change in the beginning; however, by day 15, they followed the swelling trend of the 14 µm populations. As supported by Langer group data, the onset of swelling is pH-induced and the extent of swelling may be due to the volume of the acidic core region, which depends on microparticle size. The larger the acidic volume, the greater the swelling. After a period of 30 days, microparticle diameter begins to decrease most likely because the polymer has eroded internally to a point where the space between the polymer chains cannot be sustained and the system begins to collapse.

This study sheds light into understanding the limitations of PLGA as a delivery system by presenting a swelling profile of unmodified, monodisperse, drug-free PLGA microparticles. Multiple datasets were used to answer questions of swelling index, onset, size dependency, and reproducibility. Our study presents a timeline of microparticle transformation (swelling profile), explains the possible forces behind the delayed onset of substantial swelling with the help of other corresponding studies, and provides information to help predict microparticle behavior in terms of initial size verses swelling capacity for PLGA 50:50 38,000–54,000 mw. Altogether, this study advances the understanding of PLGA microparticle limitations and potentials, and thereby benefits the drug delivery, material science, and biomedical engineering fields.

## Methods

### Materials

Poly(d,l lactic-co-glycolic acid) (PLGA, 38,000–54,000, 50:50 lactic acid: glycolic acid, acid terminated; SIGMA); Polyvinyl alcohol (PVA, average 30,000–70,000 mw, 87–90% hydrolyzed, Sigma Aldrich); High-speed FASTCAM camera (PCI-10K, Photron LTd.); high-speed Phantom monochrome camera (V310, Vision Research); Inverted microscope (Nikon); Plastic 1 ml Luer Norm-Ject syringes (Henke Sass Wolf); Glass 250 µl–1 ml Hamilton gastight instrument syringes (Hamilton); Pico Plus Syringe pumps (Harvard Apparatus); Teflon (PTFE) tubing (wall thickness 0.012, AWG 20, ICO Rally); PDMS (poly(dimethyl) siloxane, Sylgard 184, Dow Corning), Teflon AF (DuPont); Fluorinert FC-43 (3M); Biopsy punches (Integra Miltex); Air plasma chamber (Harrick Scientific); Dimethyl Carbonate (DMC, anhydrous 99%, SIGMA), PBS (Phosphate Buffered Saline, pH 7.4, without Calcium or Magnesium, Lonza).

### Fabrication of microfluidic devices

Microfluidic devices utilized in experiments were fabricated by the poly(dimethyl) siloxane (PDMS) replica molding process using standard soft lithography techniques^50^. Briefly, a silicon wafer is spin coated with SU8-2050 photoresist and soft baked according to MicroChem feature height requirements. For this work, 60 µm and 100 µm height formulas were used. A photomask containing device channel features is placed on top of the wafer, exposed to UV light, baked, and submerged into a photo developer to bring out the positive mold features. Once the photolithography process is complete the wafer is spin coated with 1% (v/v) Teflon. Uncured PDMS is mixed in a 9:1 (w/w) ratio of pre-polymer base to curing agent, poured onto the silicon wafer mold, degassed for 20 min, and cured in a 65˚C oven for a minimum of three hours. After curing, the PDMS is cut into individual devices and inlet/outlet holes are bored with 0.7–1.0 mm biopsy punches. The surface of individual PDMS devices and standard glass slides are plasma treated for 1–2 min at 300 mTorr. PDMS devices are bonded to glass slides immediately after plasma treatment and subsequently placed in a 120 °C oven for 5 min. Microfluidic channels are selectively treated to make them hydrophilic. PDMS devices must be PVA treated within several hours after plasma bonding because the PDMS is hydrophilic after plasma surface treatment and will revert to its natural hydrophobic state sometime after plasma treatment. The treatment process is done by dispensing approximately 10 µl of a 1 wt% PVA solution into the channel inlet of choice. The solution is pulled through the channel by applying a vacuum to the device outlet. The device continues to dry under vacuum suction for 2 min or more after the PVAsolution is cleared from the channel.

### Fabrication of PLGA microparticles using microfluidic devices

Microparticles are prepared from using an oil-in-water single emulsion solvent extraction process using a microfluidic droplet generator with a flow-focus design. PLGA is dissolved in non-toxic organic solvent, dimethyl carbonate (DMC). The DMC + PLGA dispersed phase solution is emulsified in a 1 wt% PVA continuous phase solution within a droplet-generating microfluidic device. The continuous and external phase channels are 50 µm in width. The flow-focusing nozzle openings for devices are 20 µm or 30 µm. A 1–10 wt% PLGA 50:50 formulation was utilized in particle formation. Microparticles are formed after the DMC solvent is extracted/diffuses from the emulsion while in aqueous solution such as PBS.

### Optical imaging and sizing

Images and videos were captured on a Phantom or high-speed FASTCAM camera mounted to a Nikon inverted microscope. Captured images of particles were analyzed in the ImageJ (NIH) image analysis program.

### In vitro swelling study

A total of 15 microcentrifuge tubes (5 sizes in triplicate) containing a 1 mL PBS, pH7.4, suspension of microparticles were incubated at 37 °C for 50 days. At different time intervals between 0 to 50 days, a 5–10 µL aliquot of microparticles was removed, placed on a glass slide, and imaged with a high-speed Phantom monochrome camera. An average of 25 microparticles were measured per aliquot. The microcentrifuge tubes were immediately returned to 37 °C once aliquots were removed. Fresh PBS was not placed into the tube after each sample was taken. After imaging, microparticles were measured with ImageJ software to determine their size.
